# The Effect of *Rosmarinus officinalis* Essential Oil Fumigation on Biochemical, Behavioral, and Physiological Parameters of *Callosobruchus maculatus*

**DOI:** 10.3390/insects11060344

**Published:** 2020-06-03

**Authors:** Michał Krzyżowski, Bartosz Baran, Bartosz Łozowski, Jacek Francikowski

**Affiliations:** Laboratory of Insect Physiology and Ethology, Institute of Biology, Biotechnology and Environmental Protection, Faculty of Natural Sciences, University of Silesia in Katowice, 40-007 Katowice, Poland; bartosz.m.baran@gmail.com (B.B.); bartosz.lozowski@us.edu.pl (B.Ł.); jacek.francikowski@us.edu.pl (J.F.)

**Keywords:** enzyme activity, stored product protection, GST, CAT, AChE, locomotor activity, repellent effect, insecticide

## Abstract

This study explores the influence of rosemary, *Rosmarinus officinalis* (L.) essential oil (EO) on the biochemical (acetylcholinesterase, catalase, and glutathione S-transferase), physiological (oxygen consumption), and behavioral (open field test, repellency) parameters of an important stored product insect: cowpea weevil, *Callosobruchus maculatus* (F.). *R. officinalis* EO exhibited effective insecticidal action against *C. maculatus* even at relatively low concentrations. LC_50_ = 15.69 μL/L air, and was highly repellent at concentrations equal to or above LC_25_. Statistically significant inhibition in locomotor activity occurred only after the acute exposure to the EO at LC_12.5_ and LC_25_. The oxygen consumption test showed metabolism increase only at LC_50_. An increase in activity was observed in the case of all three enzymes examined. The presented data provides a potentially valuable resource in designing more environmentally friendly and safer insecticide agents.

## 1. Introduction

Rosemary, *Rosmarinus officinalis* (L.), is an herbaceous member of the mint family Lamiaceae, and is native to the Mediterranean region. However, it is well-known, and globally and widely cultured because of its cultural, culinary, aesthetic, and folk medicinal usage [1]. A distinctive characteristic of *R. officinalis* is its fragrance, originating from the high content of volatile compounds in the plant material. These compounds can be extracted by steam distillation and concentrated in the form of *R. officinalis* essential oil (EO).

The evolutionary role of EOs in plants is mainly theoreticized to be a protective agent against pests, including insects and fungi. *R. officinalis* EO is known to be an effective fumigant agent against various insect pests, such as confused flour beetle, *Tribolium confusum* (du Val.) (Coleoptera: Tenebrionidae) [2], red flour beetle, *Tribolium castaneum* (Herbst) (Coleoptera: Tenebrionidae) [3], almond moth, *Cadra cautella* (Walker) (Lepidoptera: Pyralidae) [4], and pulse beetle, *Callosobruchus chinensis* (L.) (Coleoptera: Chrysomelidae) [5]. Thus, *R. officinalis* is a promising bioinsecticide agent; especially valuable when considering the widely spreading resistance to conventional insecticides [6]. Although there are relatively numerous studies on the insecticidal effectiveness of the EO against cowpea weevil, *Callosobruchus maculatus* (F.) (Coleoptera: Chrysomelidae) most of them focus exclusively on mortality assessment omitting behavioral and biochemical parameters [5,7,8,9]. The insecticidal effects of EOs are multimodal—affecting a broad range of the physiological processes.

One of the most widely recognized hypotheses of the mode of action *R. officinalis* EO toxicity is its ability to inhibit the acetylcholinesterase (AChE). AChE inhibition is also the primary effect of many insecticides, such as organophosphates and carbamates [10]. AChE is one of the key enzymes in maintaining the cholinergic transmission in insects’ central nervous system; thus, its inhibition could cause a broad spectrum of primary and secondary effects [11], especially due to the disruption of the neural transmission in head ganglia and ventral nerve cord. It is speculated that the EO’s components responsible for the AChE inhibition may be terpenes and monoterpenes, which are the main constituents of *R. officinalis* EO 1,8-cineol (monoterpenoid), camphor (terpene), and α-pinene (monoterpenoid) [12].

The main aim of the presented study was to evaluate the insecticidal potential of *R. officinalis* EO against *C. maculatus* and its influence on the biochemical (acetylcholinesterase, catalase, and glutathione S-transferase), physiological (oxygen consumption), and behavioral (open field test, repellency) parameters.

## 2. Materials and Methods

### 2.1. Insect Rearing

Cowpea weevils, *C. maculatus*, were reared on mung beans (*Vigna radiata*) ad libitum obtained from a local vendor. One-week-old, mixed-sex adults were used in the experiments. The insects were reared under constant conditions of 31 ± 1 °C, relative humidity 50 ± 10%, and the photoperiodic regime of 12:12 L:D.

### 2.2. Used Substance

The *R. officinalis* EO used in the assessments was water, distilled, and provided by local supplier Naturalne Aromaty sp. z o.o.

### 2.3. Chemical Analysis

Compounds were identified by gas chromatography-mass spectrometry (GC-MS) analysis. The mixture was separated using ZB-5ms column (Phenomenex) and then the single constituents were identified using GC-MS-QP2010 SE mass spectrometer (Shimadzu). The injection volume was 1 µL (split ratio 200:1) and the oven temperature profile was as follows: 50 °C for 2 min followed by an increase of 4 °C min^−1^ to 280 °C; 280 °C for 2 min; an increase of 40 °C min^−1^ to 320 °C; 320 °C for 5 min. Helium with the flow of 2 mL min^−1^ was used as the carrier gas.

### 2.4. Fumigation Mortality

Fumigation mortality was assessed in four replications per volume, each comprising 30 concentrations (0.1–3.0 µL by 0.1 µL increments). Each replication consisted of ten mixed-sex insects (n = 40; Σn = 1240) that were put into 50 mL non-hermetic, conical, plastic containers with tight-fitting lids (30 mm height, 55 and 48 mm diameter). Tested EO (or ultrapure water in the control group) was applied on a cotton pad attached to the cover of the container. Dead beetles were counted after 24 h. Insects were considered dead when no movement for 1 h was observed. The bioassays were conducted under constant conditions, i.e., 30 ± 1 °C, 50% relative humidity. LC_50_ was determined by fitting a model to the data obtained from the mortality assay. The model was fitted with the least-squares fit method. The LC50 was calculated from the slope of the fitted curve using GraphPad Prism v6.00 software (GraphPad Software, San Diego, CA, USA).

### 2.5. Repellency

To assess the repellent activity of the *R. officinalis* EO, a repellency test described by Francikowski et al. [13] was adapted. For every volume (volume corresponding to LC_12.5_; LC_25_; LC_50_), 40 beetles were used. Insects were placed individually in rectangular chambers (3 mm height, 15 mm width, 160 mm long, made of transparent Lucite). In each of the chambers, a flow of air from both sides was maintained. The inlet air for the odor side was pumped through the cotton wool (placed inside the gas bubbler in place of liquid) with applied EO (the volumes of applied EO have been recalculated to match the volume of the gas bubbler—200 cm^3^) while for the opposite site through cotton wool with an equal volume of ultra-pure water. The airflow was kept constant at 10 L/h. The insects were able to explore the chambers freely, and their movement was recorded with Microsoft LifeCam Studio and AMCap software. Recordings lasted for 10 min at 15 fps frame rate with 640 × 860 px resolution. The obtained data was analyzed as described in Baran et al. [14], which allowed the calculation of the Preference Index (PI).

### 2.6. Open Field Test

The locomotor response of *C. maculatus* to *R. officinalis* EO fumigation was assessed in the open field test—performed in the glass Petri dish (ø = 100 mm, h = 20 mm) arenas. The assessments were conducted in four replications per volume corresponding to LC_12.5_; LC_25_; LC_50_ (the volumes have been recalculated to match the volume of the Petri dish—157.08 cm^3^). For each replication ten mixed-sex insects were used (n = 40; Σn = 320). Open field assessments were conducted in two variants: prolonged exposition (24 h of fumigation) and acute (insect movement was recorded directly during the fumigation). The fumigation was performed in the Petri dishes lined with filter paper disks (Whatman N°1). On each paper filter, a specific volume of the EO was applied.

The locomotor activity recording lasted for 50 min. The analysis of the video was conducted in five intervals, each consisting of ten minutes. Trajectories of insects’ movement were extracted with SwissTrack 4 software [15] and subsequently analyzed in the R environment with trajr [16] package. The time spent on resting and distance traveled were analyzed.

### 2.7. Oxygen Consumption

An oxygen consumption test was conducted in four replications, using a SiLab data acquisition unit and oxygen sensor (sampling rate: 1/sec) tightly fitted into the 50 mL Falcon^®^ tube (BD Biosciences, San Jose, CA, USA). For each experiment, ten mixed-sex individuals (n = 40; Σn = 160) were put into airtight tubes containing 15 g of mung beans. To avoid the contact of the insects with the tested EO, cotton wool wetted with EO was placed in meshed three-dimensional (3D) printed containers (cylindrical, d = 20 mm, h = 26 mm fabricated using photocurable resin) mounted on the bottom of Falcon^®^ tubes. To each of the containers, the volume corresponding to LC_12.5_; LC_25_; LC_50_ of the tested EO (ultrapure water in the control group) was applied. Measurements were started immediately after putting insects into the Falcon^®^ tube and lasted for 1 h. The bioassays were conducted in constant conditions, i.e., 30 ± 1 °C, 50% relative humidity.

### 2.8. Enzyme Assays

For each replication, 10 mixed-sex insects were weighed and homogenized in Sorensen’s buffer (0.05 M; pH 7.4) in a 1:10 ratio. Thereafter, the homogenate was centrifuged (10,000 RPM, 10 min, 4 °C). Blind tests were prepared using buffers instead of homogenates. All measurements were performed with the Tecan M200 spectrophotometer (Tecan Group Ltd., Männedorf, Switzerland) in the Corning^®^ 96-well UV-Transparent microplates (Corning, Tewksbury, MA, USA). For each well, five measurements in different regions were taken, and the obtained results were averaged. In the samples the protein content was determined using the Bradford method, and then the enzyme activity was converted into Δ/min/mg of protein [17].

#### 2.8.1. Acetylcholinesterase (AChE) Activity Assay

The AChE activity was determined by the colorimetric method of Ellman et al. [18], based on the changes in the absorbance of 412 nm light by DTNB (Ellman’s reagent) over time in the presence of AChE. The reaction mixture consists of 150 μL DTNB (0.01 M), 20 μL AChTI (0.075 M), 10 μL probe. The eight consecutive measurements were performed every 30 s.

#### 2.8.2. Catalase (CAT) Activity Assay

The catalase activity was determined using a modified Orr’s [19] spectrophotometric assay based on the changes of the absorbance of 230 nm UV light by H_2_O_2_ over time in the presence of catalase. The samples were diluted 40 fold in Sorensen’s medium (0.05 M; pH 7.4). The reaction mixture consists of 40 μL 0.03 M H_2_O_2_ and 80 μL of the diluted sample. The six consecutive measurements were performed every 10 s.

#### 2.8.3. Glutathione S-Transferase (GST) Activity Assay

The GST activity was determined using Yu’s spectrophotometric assay [20] based on the changes of the absorbance of 340 nm light by CDNB (GST substrate) over time in the presence of GST. The reaction mixture consists of 10 μL of the sample, 5 μL CDNB (15 mM), and 200 μL GSH (1 mM). The six consecutive measurements were performed every 30 s.

### 2.9. Statistical Analysis

All statistical analysis were conducted using GraphPad Prism v6.00 software on Windows OS. For all the obtained data, the normality test was performed (Shapiro–Wilk normality test). The repellent effect was analyzed with the usage of a nonparametric test—Kruskal–Wallis with multiple comparisons. For the analysis of oxygen consumption, locomotor activity, and enzymatic activity, an ANOVA multiple comparisons test (Tukey test, Holm–Sidak test, *p* < 0.05) was used. Additionally, for the locomotor activity the Two-way RM (reapeted measures) ANOVA was performed with *p* < 0.05.

## 3. Results

### 3.1. Chemical Analysis

The GC-MS analysis revealed that the main constituents of the examined EO were α-Pinene (22.64%), Camphor (21.84%) and 1,8-Cineole (21.53%) (Table 1).

### 3.2. Mortality

**Figure 1:**
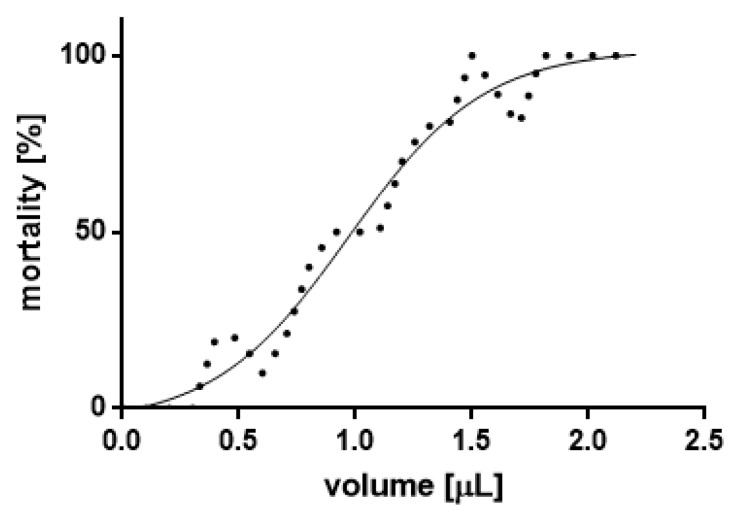
Model determining mortality curve, based on obtained mortality data (LC_50_ 0.9709 μL/50 mL, slope 1.495, degrees of freedom 65, R^2^ 0.9614).

Based on the results of the mortality assessment (Figure 1), a model was developed to determine the concentrations used in further assays (LC_12.5_, LC_25_, LC_50_). Subsequently, LC_12.5_ = 0.4132 μL/50 mL, LC_25_ = 0.6518 μL/50 mL, LC_50_ = 0.9709 μL/50 mL.

### 3.3. Repellency

**Figure 2:**
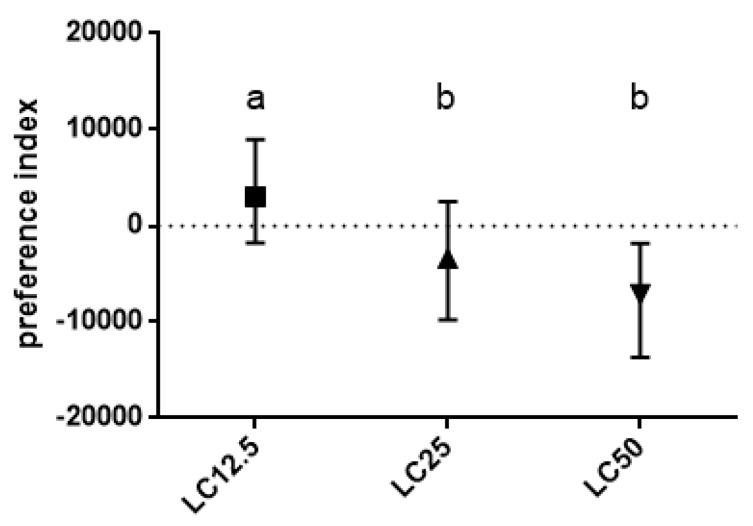
Preference index (PI) for adult insects treated with three concentrations (LC_12.5_, LC_25_, LC_50_) of *R. officinalis* EO. Median and quartiles values are presented. Letters indicate statistically homogenous groups. N = 40, Kruskal–Wallis with multiple comparisons, *p* < 0.05; treatment effect: K–W value 21.54, *p* < 0.0001.

The results of the preference assay using three determined concentrations (LC_12.5_, LC_25_, LC_50_) showed differentiated reactions depending on the used concentration (Figure 2). For insects treated with LC_12.5_ of *R. officinalis* EO, the PI was close to zero. However, groups treated with LC_25_ and LC_50_ differed significantly from the LC_12.5_ group. The repellent effects of EO in the groups treated with LC_50_ are demonstrated by the insects avoiding the region of the system with odor inlet.

### 3.4. Open Field Test

**Figure 3:**
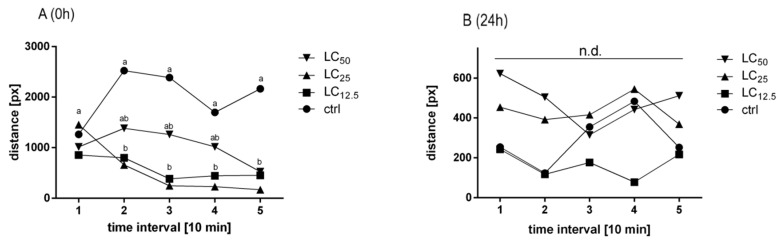
Distance traveled by the adult insects treated with three concentrations (LC_12.5_, LC_25_, LC_50_) of *R. officinalis* EO, direct (**A**) and 24 h (**B**) after the exposure. Mean values in every interval are presented. Letters indicate statistically homogenous groups in a particular time interval (10 min). N = 4, ANOVA, Tukey’s multiple comparison test, *p* < 0.05; treatment effect: (A) F (3, 12) = 8.951, *p* = 0.0022; (B) F (3, 12) = 1.648, *p* = 0.2306.

The effect of *R. officinalis* EO on the locomotor activity of the *C. maculatus* varied significantly depending on the time between treatment and measurement. Immediately after the treatment, the activity measured in the first interval is comparable in all the groups. The differences emerged with the second interval where insects treated with LC_12.5_ and LC_25_ displayed a significant decrease in activity in comparison to control. The group treated with LC_50_ displayed activity at the level of between the control and groups treated with lower concentrations but did not differ from those groups until the fourth interval. LC_50_ differed significantly from the control in the fifth interval, where the activity decreased to the level of the groups treated with lower concentrations. Activity after 24 h was significantly lower, and the groups did not differ from each other (Figure 3) throughout the measurement period.

### 3.5. Oxygen Consumption

**Figure 4:**
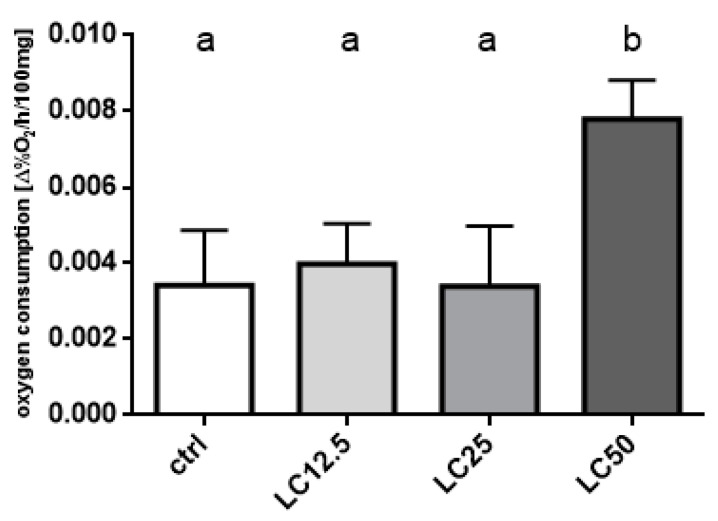
Level of oxygen consumption (mean ± SD) by insects treated with three concentrations (LC_12.5_, LC_25_, LC_50_) of *R. officinalis* EO for 1 h. Letters indicate statistically homogenous groups. N = 6, ANOVA, Holm–Sidak multiple comparison test, *p* < 0.05; F (3, 20) = 15.69, *p* < 0.0001.

The treatment with *R. officinalis* EO significantly affected the level of oxygen consumption of the exposed insects. The treatment with LC_50_ resulted in a significant increase in oxygen consumption, while groups treated with LC_12.5_ and LC_25_ did not differ significantly from the controls (Figure 4).

### 3.6. Enzyme Assays

#### 3.6.1. Acetylcholinesterase (AChE) Activity Assay

**Figure 5:**
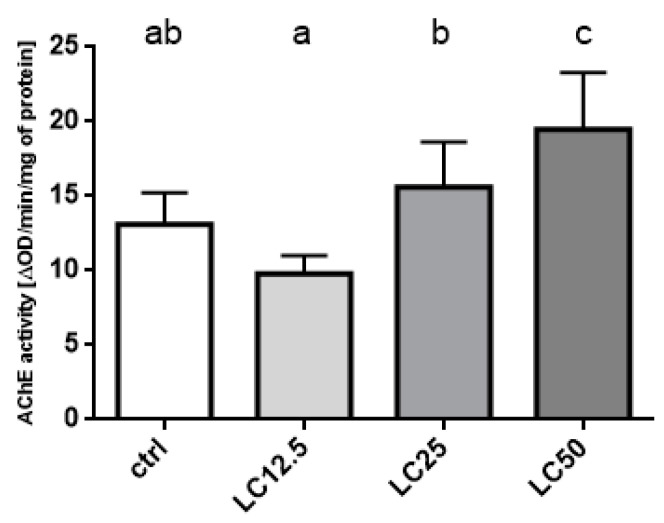
Effect of three concentrations (LC_12.5_, LC_25_, LC_50_) of *R. officinalis* EO on the activity of the Acetylcholinesterase (AChE) enzyme. Mean ± SD; letters indicate statistically homogenous groups. N = 6, ANOVA Holm–Sidak multiple comparison test *p* < 0.05; F (3, 20) = 13.58, *p* < 0.0001.

The analysis of acetylcholinesterase activity revealed changes caused by the treatment with *R. officinalis* EO. The two lowest LC_12.5_ and LC_25_ concentrations did not differ significantly from the control (however, differed significantly from each other), while the strongest stimulation of activity was observed again in the group treated with LC_50_ (Figure 5).

#### 3.6.2. Catalase (CAT) Activity Assay

**Figure 6:**
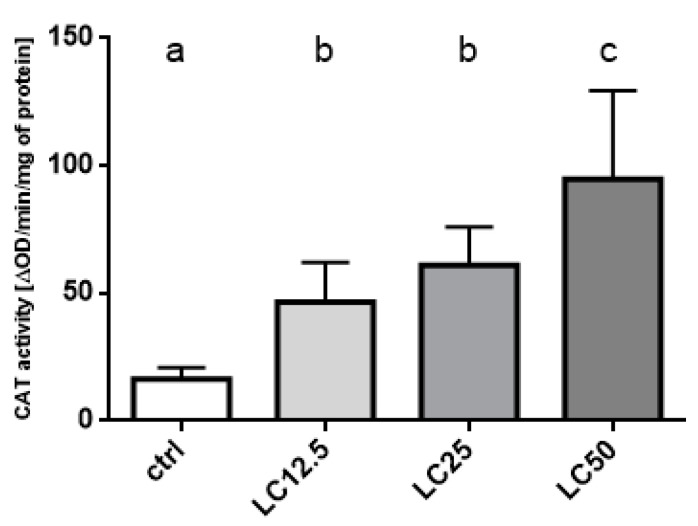
Effect of three concentrations (LC_12.5_, LC_25_, LC_50_) of *R. officinalis* EO on the activity of the catalase (CAT) enzyme. Mean ± SD; letters indicate statistically homogenous groups. N = 6, ANOVA Holm–Sidak multiple comparison test *p* < 0.05; F (3, 20) = 15.10, *p* < 0.0001.

Catalase activity in the control group was the lowest of all groups. Increased concentration of EO increased catalase activity. The groups treated with LC_12.5_ and LC_25_ did not differ from each other, but they differed from the control and LC50 groups. The highest activity was observed in the group treated with LC_50_ concentration (Figure 6).

#### 3.6.3. Glutathione S-Transferase (GST) Activity Assay

**Figure 7:**
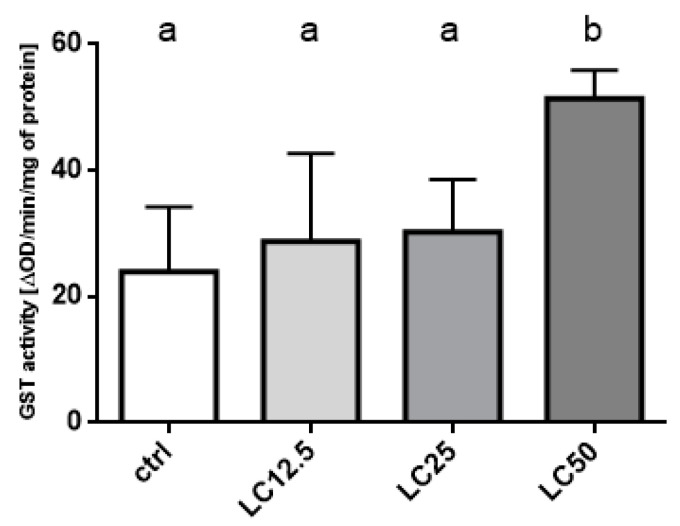
Effect of three concentrations (LC_12.5_, LC_25_, LC_50_) of *R. officinalis* EO on the activity of the Glutathione S-transferase (GST) enzyme. Mean ± SD; letters indicate statistically homogenous groups. N = 6, ANOVA Holm–Sidak multiple comparison test *p* < 0.05; F (3, 20) = 9.180, *p* = 0.0005.

The Glutathione S-transferase activity did not change in the two lowest applied concentrations (LC_12.5_ and LC_25_), compared to, the control group. Significant differences were observed only in the group treated with LC_50_ concentration compared to control (Figure 7).

## 4. Discussion

Contemporary agriculture relies heavily on pesticides, including insecticides. However, due to their extensive (and frequently inappropriate) usage, these substances are permeating into soil and ground waters, posing a risk to non-target organisms, including humans [22]. Therefore, there is a growing emphasis on developing safe alternatives that could potentially replace commonly used insecticides, while also being cheaper and more convenient to use.

One group of substances possibly possessing required insecticide characteristics to which increasing attention is being given are volatile plant extracts—essential oils. Their insecticidal activity, biodegradability by the soil microorganisms, and low toxicity to mammals, make them a good candidate as suitable pest management agents [23]. Moreover, many of these substances have been declared as generally recognized as safe (GRAS) by the United States Food and Drug Administration and are widely used in the food industry as flavoring agents [24].

Based on a wide range of studies and pilot tests conducted by authors, herein the *R. officinalis* EO presented high insecticidal potential against *C. maculatus* is an extract from *R. officinalis*. In a fumigant mortality assay, we showed that LC_50_ of this EO against *C. maculatus* is 19.4 μL/L air. The presented data coalesce with previously reported results published by Gudek and Cetin [7], where the LC50 value was 15.69 μL/L air, and support the potential of the application of the said EO as the pest management agent. In addition, they demonstrated that *R. officinalis* EO does not affect the germination capacity of protected commodities [4].

Most of available articles on insecticidal potential of EOs focus mainly on the mortality assessments or EOs’ influence on biochemical parameters, while the presented article aims to examine the influence of the rosemary EO also on physiological and behavioral parameters. Analysis of a broad spectrum of parameters allows a better understanding of the influence of EO on the tested insect.

Repellency is a behavioral measure describing the extent of the aversive action of the tested substance on the tested organism. Stronger the repellent action—the further the extent to which the animal would avoid the space treated with the tested substance. Most of the commonly used insecticides, e.g., pyrethroids, do not exhibit such action, acting solely as biocides. This characteristic, while in some situations beneficial, renders them (if used alone) inadequate for integrated pest management; therefore analyzing possible repellent activity is crucial for designing pull–push systems. *R. officinalis* has been reported to possess a repellent effect against many insect species [13]. Similar results were observed in the presented study as the *R. officinalis* were found to significantly repel the insects in the concentration equal to or higher than LC_12.5_; the repellency observed after the exposition to LC_50_, was stronger albeit, insignificantly. This result could potentially relate to another behavioral parameter analyzed, the locomotor activity, which provides a more generalized overview of baseline insect activity. The locomotor activity measured in the open field paradigm is affected not only by external stimuli as it is analyzed in the case of repellency assay but also in an internal state of the insects’ physiology. We observed significant inhibition of insects’ locomotion, directly after the exposition, in all treated groups except the one treated with LC_50_. Therefore, the observed repellent effect could not be straightforwardly explained by inhibition of movement but the active avoidance of the region with the vapors of *R. officinalis* EO. The significantly lowered locomotor activity in treated groups may indicate various underlying effects, from the general sedation, through the disruption of gait (which could be further corroborated by observations (Video S1) to the breakage of connectivity synapses (either neuro neuronal, neuromuscular or both). Nevertheless, at baseline conditions, the lowered locomotor activity should result in decreased oxygen consumption; however, the contrary was observed, as oxygen consumption was either comparable to or elevated in comparison to the control. Such results suggest that while the insects were not involved in walking, they may have been exhibiting spontaneous muscle contractions or other metabolically intensive processes related to the EO action (Video S1). In contrary to the activity directly after the exposition, no statistically significant differences between the examined groups were observed after 24 h. This may be since only alive insects were used in the assessment. This indicates that those individuals belonged to a subpopulation that effectively manages exposure to *R. officinalis* EO.

One of the potential mechanisms underlying the observed reactions in insects caused by the rosemary EO could be AChE inhibition [25]. The changes in its activity cause the disruption of the cholinergic transmission in head ganglia and ventral nerve cord causing uncoordinated legs movement [10]. Numerous articles describing the insecticidal effect of EO also point to the AChE inhibitory effect [26,27,28,29,30]. The same effect was reported by Abdelgaleil at al. (2009) where the influence of various monoterpenes was tested on the *Sitophilus oryzae* and *Tribolium castaneum* [26]. One of the substances that has inhibited enzymatic activity the most was 1,8-cineole. Additionally it was reported that the two major constituents of *R. officinalis* EO—aforementioned 1,8-cineole and camphor (Table 2) act synergistically with each other. The potent insecticidal effect (against the cabbage looper, *Trichoplusia ni*, (Hübner) (Lepidoptera: Noctuidae)) of both substances combined were attributed to the enhancement of the cuticular permeability. Such a mechanism is most relevant in regards to the apical delivery; however, similar effects (increased absorption on the tracheae level) could also occur in the case of fumigation with said substances [31].

However, such results were mainly reported on *Sitophilus* species, and the assays were conducted exclusively in vitro, while the presented experiments herein were performed on living insects (in vivo). Additionally, contrary to the results in the aforementioned articles, we observed increased AChE activity in insects exposed to the EO. Such a result may be associated with the experimental design, where the enzyme activity was assessed after 24 h using only the live insects (dead individuals were excluded from the experiment because of possible post mortem enzyme degradation). This may have resulted in the assessment being carried out only on the insects that had high initial enzyme activity levels and/or were able to adapt physiologically.

The primary line of defense of insects against xenobiotics includes the CAT and GST enzymes [32]. The elevated activity levels of these enzymes are characteristic of various insect strains resistant to a wide range of insecticides. The main functions of the aforementioned enzymes include metabolizing the xenobiotics (to reduce their toxicity or to increase their solubility in water) or reduce oxidative stress caused by the accumulation of free radicals. Moreover, in case of the toxic effects of EOs, the abovementioned enzymes are involved in the defense mechanisms. Depending on the tested EOs, a decrease, increase, or no changes in activity was reported in available studies [33,34,35,36]. The reduction of activity may indicate the inhibitory activity of the EOs’ components. This may imply that the composition of the EO and, thus, the action of these components on the biochemical level, will differentiate the reactions on the level of detoxification enzymes. In the presented experiments, an increase in the activity of both enzymes was observed. Additionally, the changes in CAT and GST activity indicate the occurrence of oxidative stress as a result of EO exposition [32]. Differences in the change in activity, depending on the applied EO concentration, indicate higher sensitivity of CAT than GST (statistically significant increase in activity of the former at lower concentration: LC_12.5_). This may also be due to the fact that free radicals appear earlier than the xenobiotic itself at a concentration high enough to activate GST. The results of changes in activity combined with oxygen consumption indicate a complex multi-aspect metabolic and biochemical response of the insect to contact with rosemary EO.

## 5. Conclusions

The present study demonstrates the toxicity of R. officinalis EO against C. maculatus and its influence on the selected physiological, biochemical, and behavioral parameters. The tested EO exhibited significant repellency in concentrations equal to or exceeding LC_25_. Additionally, we observed an increase of activity of all tested enzymes and oxygen consumption in LC_50_ concentration. However, the locomotor activity was initially inhibited, but after the 24 h, the effect reverted to the baseline levels. The observed effects may be caused by the action of the main constituents of the EO: 1,8-cineol, camphor, and α-pinene. The acquisition of in-depth knowledge on how the EOs affect insect organisms could allow for the more informed approach in designing precisely targeted insecticidal agents (i.e., designing composite insecticide formulations with inhibitors of detoxification enzymes) and, thus, provide a valuable weapon in the fight against the development of resistance.

## Figures and Tables

**Table 1:** Chemical composition of the *R. officinalis* essential oil.

Component	RI	RI Lit. *	%
Camphene	943	954	9.18
β-Pinene	943	979	5.53
α-Pinene	948	936	22.64
β-Myrcene	958	990	1.27
D-Limonene	1018	1029	4.91
o-Cymene	1042	1026	2.76
1,8-Cineole	1059	1031	21.53
Linalool	1082	1096	0.87
Camphor	1121	1146	21.84
Isoborneol	1138	1160	1.17
endo-Borneol	1138	1169	2.15
α-Terpineol	1143	1188	1.92
Bornyl acetate	1277	1288	2.45
α-Terpinyl acetate	1333	1349	0.40
Caryophyllene	1494	1419	1.32
Humulene	1579	1608	0.07

* RI (retention index) value taken from Adams (2007) [21].

**Table 2:** Lethal concentration (LC) based on obtained mortality data and parameters describing the fitness of the model.

LC (µL/50 mL) Values for Imago			
24 h		Goodness of Fit	
LC_5_	0.1157	Df	65
LC_12.5_	0.4132	R^2^	0.9614
LC_25_	0.6518	Square error	7.779
LC_50_	0.9709	Sum of Squares	3933
LC_75_	1.29		
LC_95_	1.826		

## Supplementary Materials

The following are available online at https://www.mdpi.com/2075-4450/11/6/344/s1, Video S1: behavior of *C. maculatus* individuals after the exposition to *R. officinalis* EO for respectively: 0, 15, 25 (min).
